# Heterogenous Epoxidation of Isobutene Selectively Enabled by MoSe_2_ in Hexafluoroisopropanol (HFIP)

**DOI:** 10.3390/molecules29245844

**Published:** 2024-12-11

**Authors:** Xiaodao Liang, Chenghao Zhang, Yaorong He, Yanxiong Fang, Hongyu Chen, Hongbing Ji, Yan Yang

**Affiliations:** 1 School of Chemical Engineering and Light Industry, Guangdong University of Technology, Guangzhou 510006, China; 1111906004@mail2.gdut.edu.cn (X.L.); fangyx@gdut.edu.cn (Y.F.); 2 School of Chemical Engineering, Guangdong University of Petrochemical Technology, Maoming 525000, China; 3 Guangxi Key Laboratory of Clean Pulp & Papermaking and Pollution Control, School of Light Industrial and Food Engineering, Guangxi University, Nanning 530004, China; 2316391071@st.gxu.edu.cn; 4 Key Laboratory of Bioinorganic and Synthetic Chemistry of Ministry of Education, Fine Chemical Industry Research Institute, School of Chemistry, Sun Yat-Sen University, Guangzhou 510275, China; heyr9@mail2.sysu.edu.cn; 5 Guangdong Provincial Laboratory of Chemistry and Fine Chemical Engineering Jieyang Center, Jieyang 515200, China; 6 State Key Laboratory Breeding Base of Green-Chemical Synthesis Technology, Institute of Green Petroleum Processing and Light Hydrocarbon Conversion, College of Chemical Engineering, Zhejiang University of Technology, Hangzhou 310014, China

**Keywords:** olefin epoxidation, MoSe_2_, hexafluoroisopropanol, hydrogen bonding catalysis

## Abstract

Herein, a high selective epoxidation of isobutene was achieved by heterogeneously dispersed MoSe_2_ with tert-butyl hydroperoxide (TBHP), which further showed versatile substrate scopes and well-retained activity among recycling tests. A rational mechanism is proposed based on extensive control experiments and electron paramagnetic resonance spectroscopy, surprisingly unveiling the metal–oxo and radical mediated pathways dramatically accelerated by hydrogen bonds of hexafluoroisopropanol (HFIP).

## 1. Introduction

Epoxides, as the cornerstone of petrochemicals, are widely applied in our daily life, especially diverse polymers [1,2,3]. The use of olefins as substrates for epoxidation has been an important method for producing epoxides. The epoxidation of metal catalysts with milder oxidants, such as O_2_, H_2_O_2_, and alkyl hydroperoxides, has led to the development of high-efficiency catalysts, which has been a major focus of research [4]. Molybdenum, being rich and inexpensive, exhibits excellent catalytic activity, Lewis acidity, and low oxidation potential at the highest oxidation state. It also has higher activity compared to tungsten, titanium, and vanadium, making it highly desirable for researchers [1]. Various types of catalysts with very high epoxidation efficiency have been developed, including homogeneous catalysts, such as molybdenum acetylacetone [5,6] and Mo-phthalocyanine [7], as well as heterogeneous catalysts, like molybdenum-based polyoxometalate (POM) catalysts [8,9], metal–organic frameworks (MOF_S_) [10,11,12], inorganic material-supported molybdenum-based catalysts [13], and other new molybdenum-based catalysts that are emerging [14,15]. Liang et al. successfully prepared a Mo-based catalyst (Mo/CBC) using a hydrothermal method, where the carbon support was derived from agricultural waste, which is a renewable resource that contributes to sustainable catalyst development [16]. A novel heterogeneous silica-coated magnetic nanoparticle catalyst (SCMNPs-S-COO-MoO_2_) was developed by Masteri Farahani, demonstrating significant potential for enhancing the selectivity and efficiency of olefin epoxidation reactions [14]. This catalyst efficiently facilitates the addition of electron-deficient peroxide oxygen to the C=C double bonds of olefins. Homogeneous catalysts are highly dispersed in the system, which enhances the efficiency of the epoxidation reaction. However, they are difficult to separate, and recovering and recycling the catalyst poses challenges. In contrast, heterogeneous catalysts can address these separation issues, but they encounter problems such as catalyst inactivation, preparation difficulties, and low reactivity. Therefore, the effective binding of homogeneous active sites to heterogeneous support catalysts has become crucial for the epoxidation reaction of olefins. However, the molybdenum catalyst needs to operate at high temperatures to generate peroxy radicals from TBHP and activate the C=C double bond [4,8]. Given the synergistic effect between molybdenum catalysts and TBHP, we began to consider whether we could promote the epoxidation of alkenes by both at lower temperatures.

Molybdenum(IV) selenide(MoSe_2_), a novel and fascinating transition-metal dichalcogenide (TMDC) compound, is mainly utilized in electrochemistry, photocatalysis, and energy storage [17,18,19]. MoSe_2_ is a two-dimensional layered molybdenum-based sulfide with a typical graphene-like structure, comprising Se-Mo-Se molecular layers held together by a weak van der Waals force, with Mo atoms situated between two Se atoms and Mo-Mo bonded by covalent bonds. The small band gap at the Fermi level (1.29 eV) makes it easily excitable and holds promising applications in photocatalysis [20,21]. However, the catalytic role of MoSe_2_ in hydrothermal oxidation has been overlooked. Herein, we first tried to extend the fascinating applications of MoSe_2_ to heterogeneously catalyze efficient non-photo epoxidation, which selectively conversed isobutene (IB) to isobutylene oxide (IBO) with TBHP (conv. 94.6% and select. 94.2%). The versatile procedure enabled facile access to epoxidations of diverse alkenes, with excellent recycling stability up to 9 times. Further control experiments with alternative metal cations (Cr, Ni, Co, and W) and chalcogen anions (O, S, and Te) were performed, as well as electron paramagnetic resonances, surprisingly unveiling two distinct pathways mediated by the metal–oxo and radical. Both of them were dramatically accelerated by hydrogen bonds of hexafluoroisopropanol (HFIP). This is indeed interesting and prompts us to think deeply about the role of HFIP in molybdenum catalysts and the pathways mediated by metal–oxo and radicals in catalytic systems.

## 2. Results and Discussion

### 2.1. The Scopes of Catalysts

We initially introduced MoSe_2_ to catalyze isobutene epoxidation as a model reaction, showing an excellent 94.6% conversion and 94.2% IBO selectivity under mild conditions (Table 1, entry 2), in contrast to the control reaction without MoSe_2_ (Table 1, entry 1). When MoS_2_ was introduced as the alternative catalyst, there was no IBO, but there was a mixture of acids and ethers, including 3,3-Dimethylbutyric acid, methyl tert-butyl ether, isobutylene dimer, and isobutylene trimer (Table 1, entry 3). As for MoTe_2_, the conversion of IB was remarkably lowered to 59.9%, along with 95.4% IBO selectivity (Table 1, entry 4). We also examined the MoO_2_ and MoO_3_ from the commercial source (Table 1, entry 5–6), both of which exhibited quite low conversions of IB as the performance of MoTe_2_. Thus, selenium may be the best choice for the anion part of catalysts. Then, we further screened the vital effect of the metal cation, such as WSe_2_, CrSe, NiSe, and CoSe. The results showed poor conversions (15.6~44.1%) and selectivities (64.9~83.9%) (Table 1, entry 7–10).

Subsequently, the loading of the desired MoSe_2_ was further optimized by varying the amount from 0 to 15 mol%, as shown in Figure S1. Gratifyingly, the conversion of IB reached 41.6% with only 0.25 mol% catalyst loading, which dramatically increased to 2.4 times as high as that of initial 0 mol% catalyst loading. This clearly suggests that the MoSe_2_ may accelerate the transformation of the active oxygen atom of TBHP to IB. In addition, the selectivity of IBO slightly decreased from 99.2% to 89.3% along with the increasing catalyst loading. Therefore, the best loading of MoSe_2_ was confirmed as 5 mol%, with 94.6% IB conversion and 94.2% IBO selectivity.

### 2.2. The Scopes of Solvents

To discern the solvent effect during epoxidations of IB, we first investigated the catalytic performance within general solvents, such as tetrahydrofuran (THF), isopropanol (IPA), tert-Butanol (TBA), acetonitrile (ACN), 1,4-dioxane (DO), decane (DC), toluene (TL), dichloromethane (DCM), dichloroethane (DCE), ethyl acetate (EA), N, N-dimethylformamide (DMF), and 2,2,2-trifluoroethanol (TFA) (see Figure S2 for details). However, the results showed poor epoxidation efficiency, except three in halogenated solvents (DCE, DCM, and TFA), around 48.3~57.5% IB conversions and 91.7~93.7% IBO selectivities.

According to previous reports [2], the stability of peroxo intermediates composed by catalysts and peroxide is vital for epoxidation, usually relying on the intermolecular van der Waals interactions from bulk solvents, such as hydrogen and halogen. This might be an explanation for the relatively better results within halogenated solvents, and also the significant increase in the yield of IBO in HFIP [22,23,24].

### 2.3. The Effect of TBHP Dosages

Considering the stability of peroxides and the possible hydrolysis of IBO, TBHP was an optimal choice, featuring better thermal stability (151.0 ± 2 °C) in comparison with that of H_2_O_2_ (50 °C) [25,26]. TBHP in decane was commercially available and showed excellent IBO selectivity and IB conversion with 1 equivalent loading (5.5 mol/L in decane, 1.8 mL) at 65 °C [27]. (Figure S3). The water solution of TBHP under the same condition was also examined, and its IB conversion was remarkably lower at 67.7%, although the IBO selectivity remained good at 97.2% [4]. We speculated that the introduction of water might aggravate the complex phase distributions among amphiphilic TBHP, hydrophobic IB, and MoSe_2_ powder. Owing to the small water contact angle of MoSe_2_ (~45°) [28], the water molecules could competitively occupy the surface of the catalyst, which dramatically inhibited transferring the oxygen atom from TBHP to MoSe_2_, and the following epoxidation of IB.

### 2.4. The Effect of Reaction Temperature and Time

Considering the sensitivity of TBHP to temperature, the reactions should be performed below 75 °C to avoid its vigorous decomposition. At room temperature, epoxidation seemed hardly to be triggered. Along with increased temperature, the conversion of IB reached 94.6% at the optimal 65 °C, maintaining an excellent IBO selectivity at 94.2% (Figure S4). Higher temperatures resulted in increasing amounts of by-products, such as isobutyraldehyde and the corresponding ring-opening product of IBO.

Then, we further examined the effect of reaction time, which showed the best IB conversion within 2 h (Figure S5). The prolonged reaction time had little effect on the IB conversion but slightly lowered the IBO selectivity from 95.1% to 84.2% at 2 and 8 h, respectively.

### 2.5. The Recycling Experiments

After the reactions, the catalyst was simply recycled by filtration and then washed with HFIP before the next round. As shown in Figure S6, there was no obvious difference after 9 cycles, showing the outstanding robustness of the catalytic system. In addition, X-ray photoelectron spectroscopy (XPS) was also performed to examine the possible states of MoSe_2_ during the reaction. It was found that there was no change for the Mo 3*d* and Se 3*d* spectrum of reused MoSe_2_ in comparison with those of the fresh catalyst. This possibly suggests that the active metal intermediates were quickly quenched by IB (Figure S7).

### 2.6. The Solvent Recovery Experiments

HFIP, as a magic solvent, has been widely used for diverse reactions in recent years. To examine the recyclability of HFIP in our reactions, cyclohexene was chosen as a representative substrate. After epoxidation, 0.2 g BHT was first added to quench the possible remaining TBHP [29]. Under a vacuum, 13~14 mL HFIP from 20 mL of the reaction mixture was collected at 50 °C (65~70% recovery), which was then successfully used in the next cycle (92.3% conv. and 78.6% select.) (Figure S8a). After five cycles, the recovered HFIP showed good conversions and selectivity (68.5% and 76.5%, respectively) (Figure S8b).

### 2.7. Exploration of Substrate Scopes

**Figure 1:**
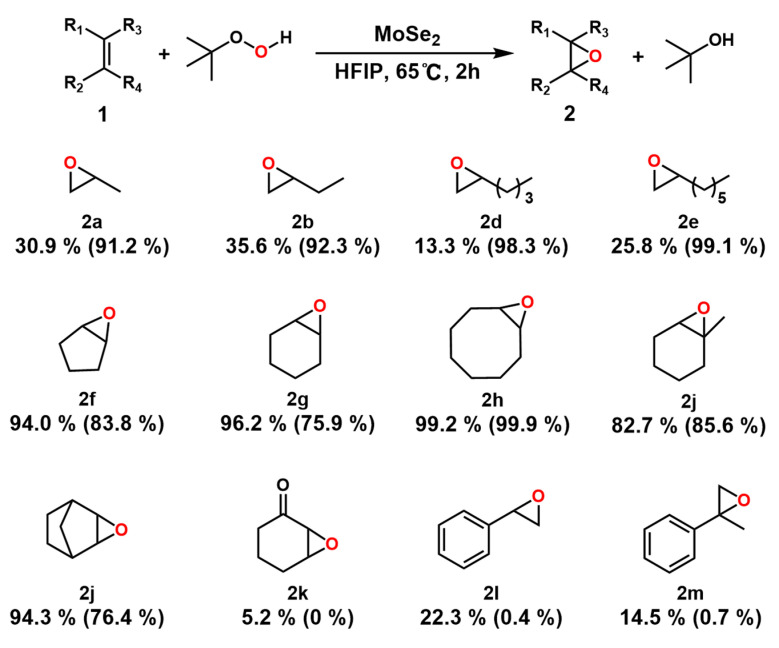
Epoxidation of different alkenes catalyzed by MoSe_2_ in HFIP. Reaction conditions: alkene (10 mmol), MoSe_2_ (5 mol%, 0.5 mmol), hexafluoroisopropanol (20 mL), TBHP (5.5 mol/L in decane, 1.8 mL) at 65 °C for 2 h. Conversion (selectivity) was determined by GC with naphthalene as internal standard.

With the optimized conditions in hands, we devoted ourselves to explore the scope of various alkenes for selective epoxidation with this unique co-catalytic system (Figure 1). The corresponding GC–MS spectra of compound **2a**–**l** are shown in Figures S10–S21. A series of alkenes with different substituted states on the C=C bonds were subjected to the epoxidations and showed good tolerance to afford the desired products **2a**–**2i** in moderate to excellent conversions and selectivities (Figure 1). The monosubstituted alkenes with the extended C1–C6 carbon chains all gave the corresponding epoxides in 13.3~35.6% conversions and 91.2~99.1% selectivities (**2a**–**d**). The cycloalkenes with varying ring sizes (*n* = 5, 6, 8) all furnished the desired cycloepoxides in outstanding yields (75.9~99.9% conv. and 94.0~99.2% select.), which is attributed to the lower activation energy of cyclo-olefins compared to that of linear olefins (**2e**–**g**) [30,31]. Especially, the substituted cycloalkenes were also selectively transformed into the corresponding epoxides, such as 1-methylcyclohexene (**2h**, 82.7% conv. and 85.6% select.) and norbornene (**2i**, 94.3% conv. and 76.4% select). However, alkenes with benzene rings and heteroatoms proved to be challenging due to the conjugation effect (**2j**–**l**) [31].

### 2.8. Proposed Mechanism for Isobutene Epoxidation

According to the previous reports, the epoxidations could be conducted via radical and metal–oxo intermediates [31,32,33]. To clarify the mechanism, we first introduced butylated hydroxytoluene (BHT) as the free radical inhibitor, which showed the obvious decreased yield of epoxied derivatives as 33.4% (in DCE, Table 2, entry 4) and 43.7% (in HFIP, Table 2, entry 5), respectively. The possible radical intermediates were further identified using electron spin-resonance spectroscopy (EPR). As shown in Figure S9, the spin-trap experiments with DMPO revealed the short-lived *t*BuOO• radical, which derived from tert-butyl hydroperoxide (TBHP) and was further confirmed by simulations (α*_N_* = 14.5, α*_N(β)_* = 10.1, *g* = 2.0061) [4]. Additionally, the corresponding EPR signal was much stronger with the introduction of MoSe_2_, consistent with the control experiments, demonstrating a remarkable catalytic performance (Table 2).

**Figure 2:**
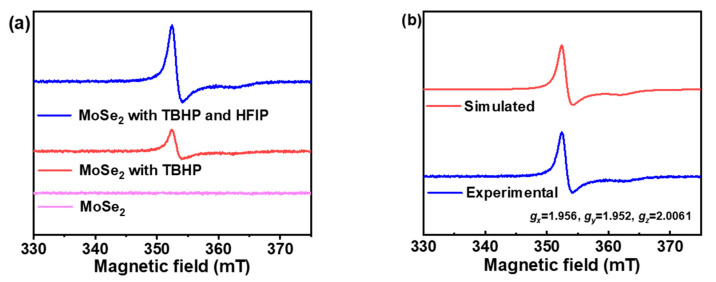
(**a**) Mo(V) EPR spectra of MoSe_2_. Purple: the referenced MoSe_2_ powder at R.T.; red: MoSe_2_ (0.5 mmol) in TBHP (1.8 mL, 5.5 mol/L in decane) at 65 °C; blue: MoSe_2_ (0.5 mmol) in mixture of TBHP (1.8 mL, 5.5 mol/L in decane) and HFIP (20 mL) at 65 °C. (**b**) EPR spectra and the corresponding simulation of MoSe_2_ (0.5 mmol) in TBHP (1.8 mL, 5.5 mol/L in decane) and HFIP (20 mL) at 65 °C.

**Scheme 1:**
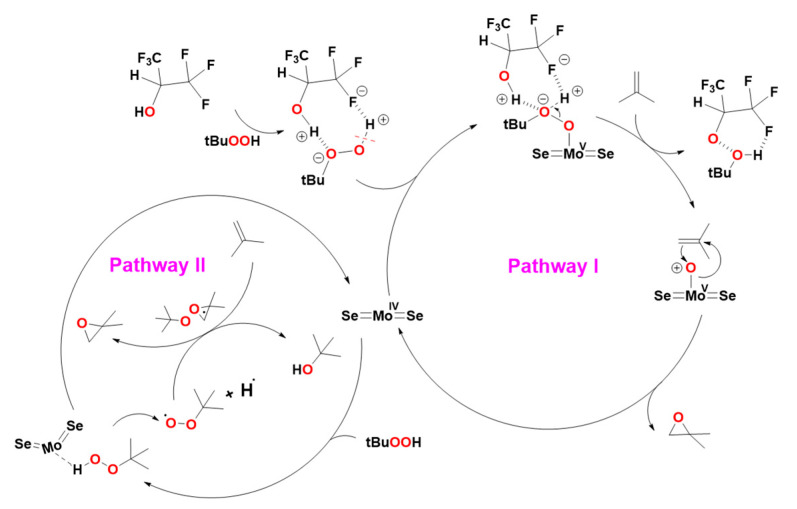
Plausible pathways of the TBHP-mediated epoxidation of isobutene catalyzed by MoSe_2_ in HFIP.

The possible metal–oxo intermediates were also determined by EPR experiments via detection of the valence of transition metals Mo with TBHP in HFIP (Figure 2a). The results revealed that the Mo(IV) in MoSe_2_ was oxidized into Mo(V) by TBHP, consistent with the corresponding simulation (*g_x_* = 1.956, *g_y_* = 1.952, *g_z_* = 1.903) (Figure 2b) [34,35]. Furthermore, the HFIP may have even facilitated the oxidation of Mo(IV) into Mo(V), according to the control experiments (blue and red lines in Figure 2a). According to previous reports, the oxygen transfer process of peroxides might be facilitated by fluoroalcohol via the polar synergistic process, where the strong hydrogen bonding interactions stabilize the transition state and lower the energy barrier of oxygen transfer significantly [36,37]. Collectively, these findings support that the epoxidation of isobutene might be realized via both radical and metal–oxo pathways, which were also dramatically accelerated by the strong solvent effect derived from the hydrogen-bonding network of HFIP. It was suggested that there were two reaction pathways in the MoSe_2_/TBHP system with HFIP, and the feasible mechanism of MoSe_2_-catalyzed epoxidation was expressed in Scheme 1. One involved the key Mo^V^-O intermediate resulting from the active oxygen transformation of TBHP to the Mo(IV) of MoSe_2_. This was facilitated by the strong electron-withdrawing properties of fluorine and the O-H hydrogen bonding characteristics. These intermediates then transferred oxygen atoms to nucleophilic olefins, transforming them into epoxides. As a result, the Mo^V^-O intermediates returned to their original state, and TBA was formed by losing the oxygen atoms of TBHP. The other pathway entailed the creation of a tert-butyl peroxide radical by TBHP, which is then followed by the epoxidation of isobutene to produce isobutylene oxide. Finally, these two pathways cooperated together to realize the efficient epoxidations of diverse alkenes under mild conditions.

## 3. Materials and Methods

### 3.1. Materials and Instrument

Isobutene was purchased form Maoming Minxing Gas Co., Ltd. (99.5%, Maoming, China). Molybdenum (IV) selenide (MoSe_2_), tungsten(IV) selenide (WSe_2_), chromium selenide (CrSe), nickel selenide (NiSe), cobalt(II) selenide (CoSe) were purchased from Aladdin Biochemical Technology Co., Ltd. (Shanghai, China); Molybdenum(IV) sulfide (MoS_2_) was purchased from Macklin Biochemical Technology Co., Ltd. (Shanghai, China); Tert-butyl hydroperoxide in decane, molybdenum trioxide (MoO_3_), molybdenum dioxide(MoO_2_), HFIP, dichloromethane, decane, tert-butanol, isopropanol, dichloromethane, dichloroethane, 2,2,2-trifluoroethanol were purchased from Anhui Zesheng Technology co., Ltd. (Anqing, Anhui, China), and *N*,*N*-dimethylformamide, tetrahydrofuran, 1,4-dioxane, ethyl acetate, acetonitrile were purchased from Shanghai Titan Scientific Co., Ltd. (Shanghai, China). All the reagents and solvents were purchased from commercial sources and used without any purification unless an anhydrous condition was noted. 

The mass spectra were obtained on a Shimadzu GCMS-2010. The GC analyses were performed on a gas phase chromatography (Nexis GC-2030, Shimadzu, Tokyo, Japan) equipped with a capillary column (Agilent Technologies CP9222 VF-WAXms, 30 m × 0.25 mm × 0.5 µm, GL Sciences Inc., Tokyo, Japan) and an FID detector (Shimadzu, Tokyo, Japan). The products were identified by comparison of their GC retention times with commercial samples.

The peroxy radicals in the ESR spectra were recorded at a microwave frequency of 9.05 GHz at 333 K by the electron spin spectrometer (JEOL Ltd., Tokyo, Japan) equipped with an X-band microwave unit. Mo(V) signals were detected by an electron spin spectrometer (Bruker A300, Karlsruhe, Germany).

The atomic structures of the catalysts were measured by an X-ray photoelectron spectroscope (Thermo Fisher, Escalab 250Xi, Waltham, MA, USA).

### 3.2. General Procedure for the Epoxidation of Isobutene

MoSe_2_ as a catalyst, TBHP in decane (5.5 mol/L) as an oxidant (if used), hexafluoroisopropanol (20 mL), and the liquid substrates were charged in the autoclave. Light olefin (C3~C4) was flushed after the autoclave was sealed. The autoclave was placed in an oil bath and heated at 65 °C for 2 h under stirring at 500 rpm. The conversions and selectivities were determined with naphthalene as the internal standard and measured by GC and GCMS. After the reaction was completed, the reactor was placed into a low-temperature reactor to cool to room temperature for about 30 min. Subsequently, the reaction solution was filtered and detected by GC.

## 4. Conclusions

The mild and selective epoxidation of isobutene catalyzed by MoSe_2_ with TBHP was successfully developed. The modular character of the catalysis provided an easy way to realize selective epoxidation of diverse alkenes with excellent yield. The possible mechanism was carefully investigated with EPR and control experiments. Optimistically, our findings unveil a new prospect of MoSe_2_-related materials.

## Figures and Tables

**Table 1:**
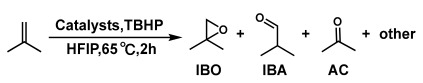
The TBHP-mediated epoxidation of isobutene catalyzed by Mo- and Se-derivatized metal salts in HFIP *^a^*.

Entry	Catalyst	Conversion(IB)/% *^b^*	Selectivity *^b^*			
			IBO	IBA	AC	Other
1	None	15.8	99.2	0.0	-	0.8
2	MoSe_2_	94.6	94.2	4.8	-	1.0
3	MoS_2_	100	-	-	-	100 *^c^*
4	MoTe_2_	59.9	95.4	2.9	-	1.7
5	MoO_2_	61.0	94.6	3.8	-	1.6
6	MoO_3_	57.9	95.2	3.0	-	1.8
7	WSe_2_	44.1	83.9	7.6	7.6	0.9
8	CrSe	15.6	64.9	6.5	24.1	4.5
9	NiSe	17.4	69.7	4.9	23.2	2.2
10	CoSe	16.4	69.0	5.0	23.1	2.9

*^a^* Reaction conditions: isobutene (0.01 mol), catalysts (5 mol%), TBHP in decane (1.8 mL, 5.5 mol/L), hexafluoroisopropanol, 20 mL), 65 °C, 2 h, in 100 mL autoclave. *^b^* Determined by GC with naphthalene as an internal standard. *^c^* The main products utilizing MoS_2_ as a catalyst were 3,3-Dimethylbutyric acid, methyl tert-butyl ether, isobutylene dimer, and isobutylene trimer. The respective selectivity rates were 56.8%, 30.1%, 8.1%, and 3.1%, with a 100% conversion rate of isobutene.

**Table 2:** Control experiments for TBHP-mediated epoxidation of isobutene *^a^*.

Entry	Derivatization from Standard Condition	IBO Yield *^b^*(%)	IBO Yield *^b^* (%)		
			IBO	IBA	Other
1	None	89.1	94.2	4.8	1.0
2	DCE as solvent	21.0	95.3	trace	4.7
3	DCE at 75 °C	45.3	98.6	trace	1.4
4	DCE with 0.1 eq BHT	33.4	98.7	1.2	0.1
5	With 0.1 eq BHT	43.7	93.2	4.0	2.8

*^a^* Standard condition: isobutene (0.01 mol), MoSe_2_ (5 mol%), TBHP in decane (1.8 mL, 5.5 mol/L), hexafluoroisopropanol (20 mL), 65 °C, 2 h. *^b^* Determined by GC with naphthalene as an internal standard.

## Data Availability

Data will be made available on request.

## Supplementary Materials

The following supporting information can be downloaded at: https://www.mdpi.com/article/10.3390/molecules29245844/s1, Figure S1: The effect of catalyst loading for the epoxidation of isobutene; Figure S2: The effect of catalyst loading for the epoxidation of isobutene; Figure S3: The effect of TBHP dosage for the epoxidation of isobutene; Figure S4: The effect of temperature for the epoxidation of isobutene; Figure S5: The effect of reaction time for the epoxidation of isobutene; Figure S6: The effect of temperature on the epoxidation of isobutene; Figure S7: (a) XPS full-scan survey of MoSe_2_. (b) XPS core-level spectra of Mo. (c) XPS core-level spectra of Se; Figure S8: (a) Amount of solvent on the solvent recovery cycles. (b) The effect of solvent recovery cycles on the epoxidation of cyclohexene. Figure S9: (a) ESR spectra of isobutene with TBHP and the spin-trapping reagent (DMPO) in DCE at 75 °C. Blue: with MoSe_2_, red: without MoSe_2_. (b) EPR spectra and the corresponding simulation of isobutene with TBHP and the spin-trapping reagent (DMPO) in DCE at 75 °C. Figures S10–S21: GC MS spectra of compound 2a~l.
