# Supplementary material for: Heterogenous Epoxidation of Isobutene Selectively Enabled by MoSe2 in Hexafluoroisopropanol (HFIP)
Source: Molecules. 2024 Dec 11;29(24):5844. doi: 10.3390/molecules29245844 (PMC11679875; doi:10.3390/molecules29245844)
Supplement: Supplementary file 1 [file molecules-29-05844-s001.zip › molecules-3301176-supplementary.pdf]

Supplementary Materials

Heterogenous epoxidation of isobutene selectively enabled by $MoSe_2$ in hexafluoroisopropanol (HFIP)

Xiaodao Liang [1, 2] , Chenghao Zhang [3] , Yaorong He [4] , Yanxiong Fang [1, 5] , Hongyu Chen [3,*] , Hongbing Ji [4,6,*] , and Yan Yang [1,*]

1 School of Chemical Engineering and Light Industry, Guangdong University of Technology, Guangzhou 510006, China

2 School of Chemical Engineering, Guangdong University of Petrochemical Technology, Maoming 525000, China

3 Guangxi Key Laboratory of Clean Pulp & Papermaking and Pollution Control, School of Light Industrial and Food Engineering, Guangxi University, Nanning 530004, China

4 Key Laboratory of Bioinorganic and Synthetic Chemistry of Ministry of Education, Fine Chemical Industry Research Institute, School of Chemistry, Sun Yat-sen University, Guangzhou 510275, China

5 Guangdong Provincial Laboratory of Chemistry and Fine Chemical Engineering Jieyang Center, Jieyang 515200, China

6 State Key Laboratory Breeding Base of Green-Chemical Synthesis Technology, Institute of Green Petroleum Processing and Light Hydrocarbon Conversion, College of Chemical Engineering, Zhejiang University of Technology, Hangzhou, 310014, China

* Correspondence: chenhongy@gxu.edu.cn; jihb@zjut.edu.cn; yangyan1209@gdut.edu.cn

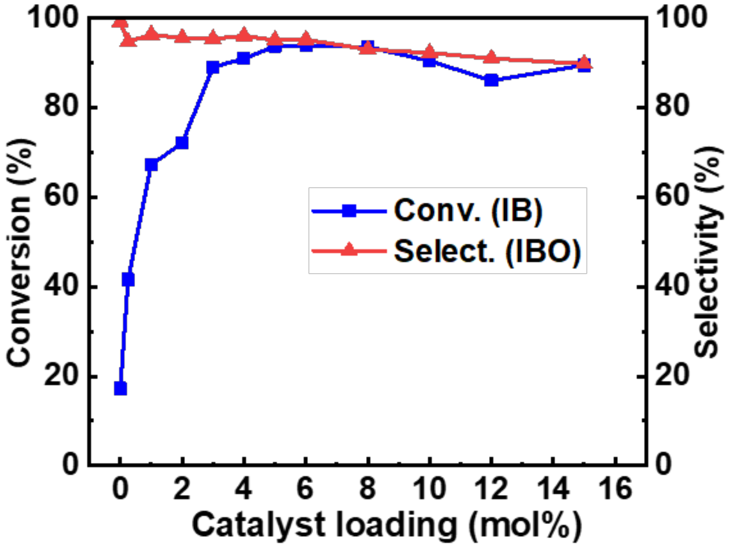

Figure S1. The effect of catalyst loading for the epoxidation of isobutene. Reaction conditions: isobutene (10 mmol), TBHP (1.8 mL, 5.5 mol/L in decane), HFIP (20 mL), 65 °C, 2 h. The conversions and selectivities were determined by GC with naphthalene as an internal standard.

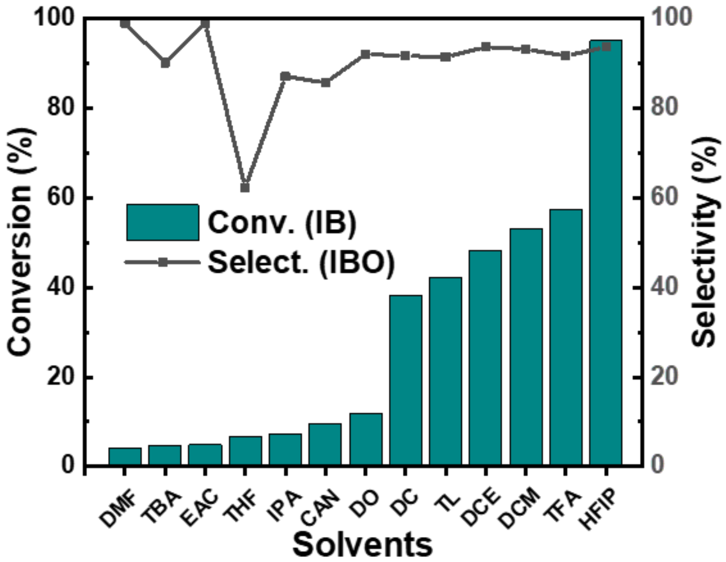

Figure S2. The Effect of solvents for the epoxidation of isobutene. Reaction conditions: isobutene (10 mmol), $MoSe_2$ (0.5 mmol), TBHP (1.8 mL, 5.5 mol/L in decane), solvent (20 mL), 65 °C, 2 h. The yields were determined by GC with naphthalene as an internal standard.

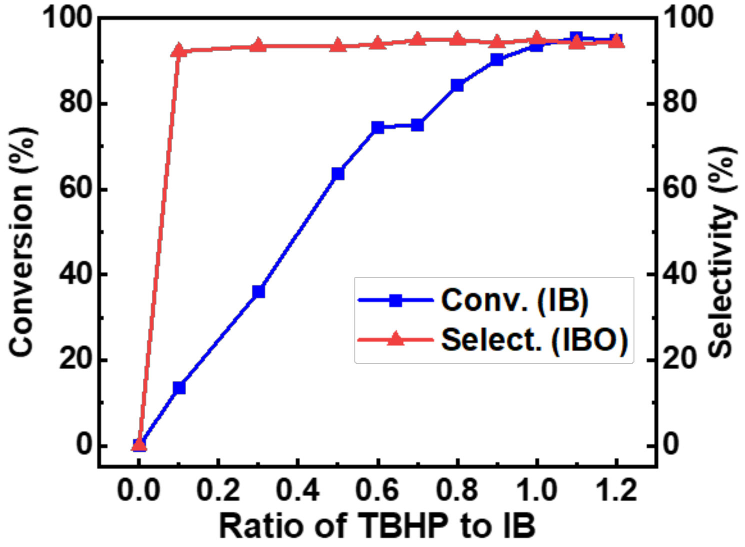

Figure S3. The effect of TBHP dosage for the epoxidation of isobutene. Reaction conditions: isobutene (10 mmol), $MoSe_2$ (0.5 mmol), TBHP (5.5 mol/L in decane), HFIP (20 mL), 65 °C, 2 h. The conversions and selectivities were determined by GC with naphthalene as an internal standard.

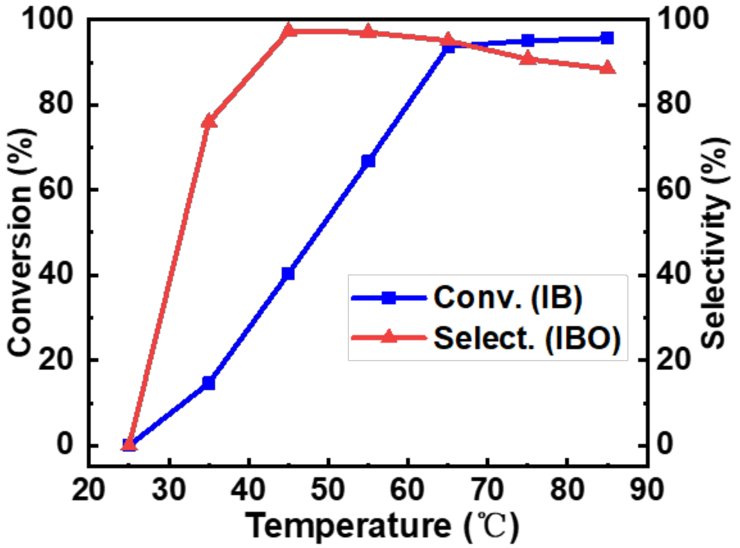

Figure S4. The effect of temperature for the epoxidation of isobutene. Reaction conditions: isobutene (10 mmol), $MoSe_2$ (0.5 mmol), TBHP (1.8 mL, 5.5 mol/L in decane), HFIP (20 mL). The conversions and selectivities were determined by GC with naphthalene as an internal standard.

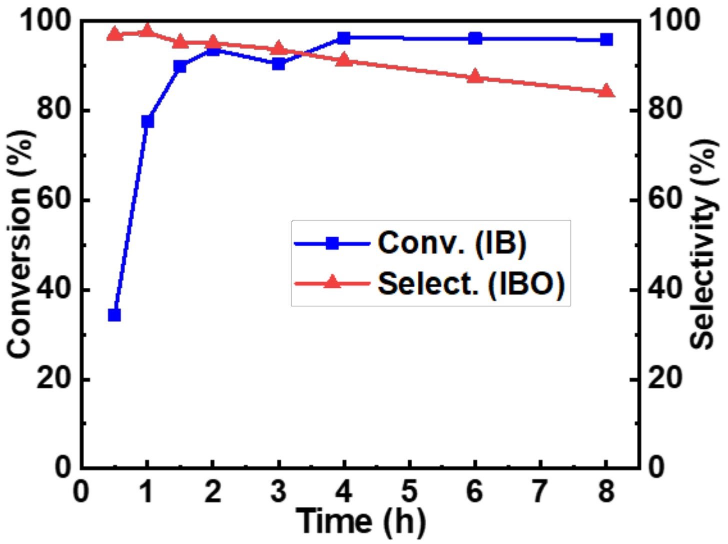

Figure S5. The effect of reaction time for the epoxidation of isobutene. Reaction conditions: isobutene (10 mmol), $MoSe_2$ (0.5 mmol), TBHP (1.8 mL, 5.5 mol/L in decane), HFIP (20 mL), 65 °C. The conversions and selectivities were determined by GC with naphthalene as an internal standard.

The reusability of $MoSe_2$ was depicted in Figure S6. After each reaction, the catalyst was filtered, cleaned briefly, dried and then proceeding with the next reaction. This process was repeated 9 times, and it was observed that the selectivity and yield of IBO showed no noticeable changes.

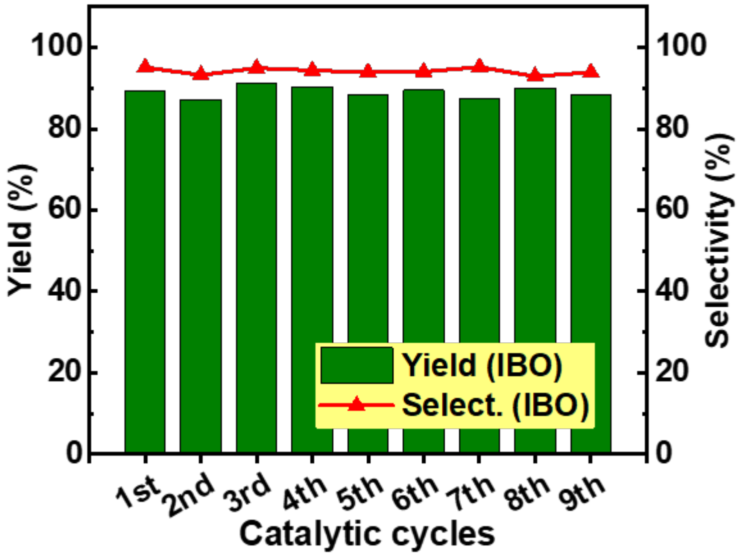

Figure S6. The effect of catalytic cycles on the epoxidation of isobutene. Reaction conditions: isobutene (10 mmol), $MoSe_2$ (0.5 mmol), TBHP in decane(1 eq, 1.8 ml), HFIP (20 mL), 65 °C， 2 h. The conversions and selectivities were determined by GC with naphthalene as an internal standard. $MoSe_2$ is recycled for 9 cycles.

As depicted in **Figure S7**, there was no alteration in $MoSe_2$ before and after the reaction, indicating the robust stability of the catalyst throughout the reaction.

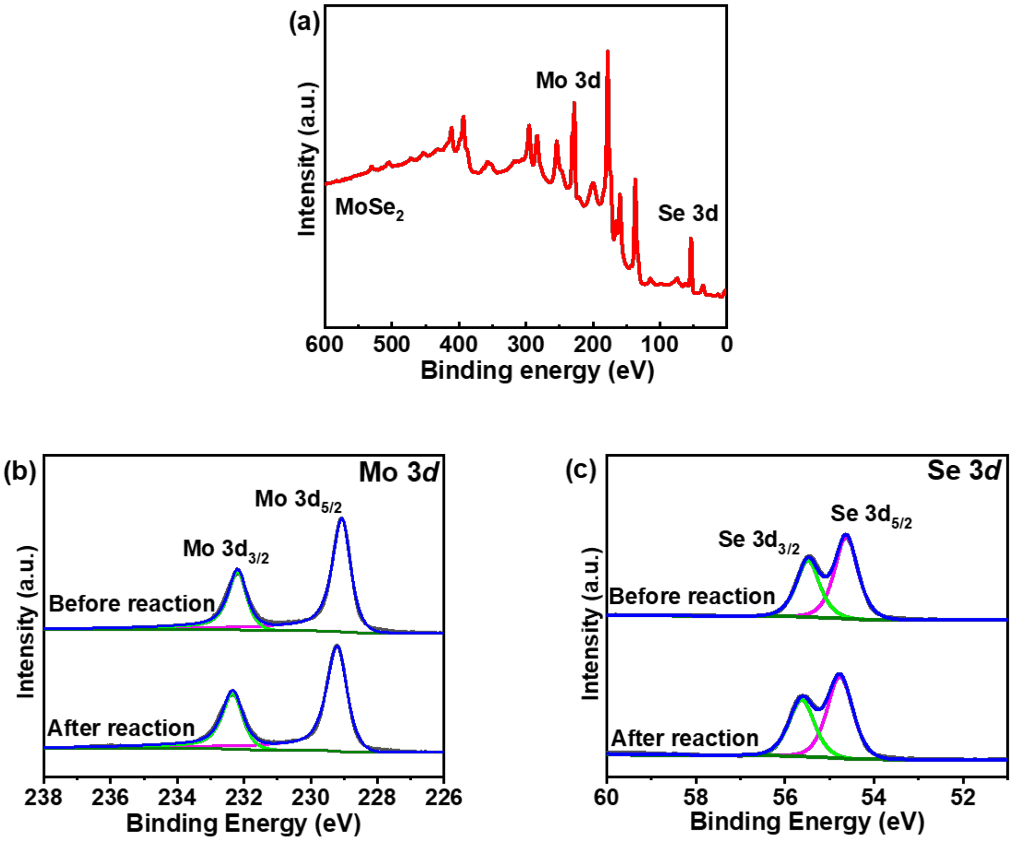

Figure S7. (a) XPS full-scan survey of $MoSe_2$. (b) XPS core-level spectra of Mo. (c) XPS core-level spectra of Se.

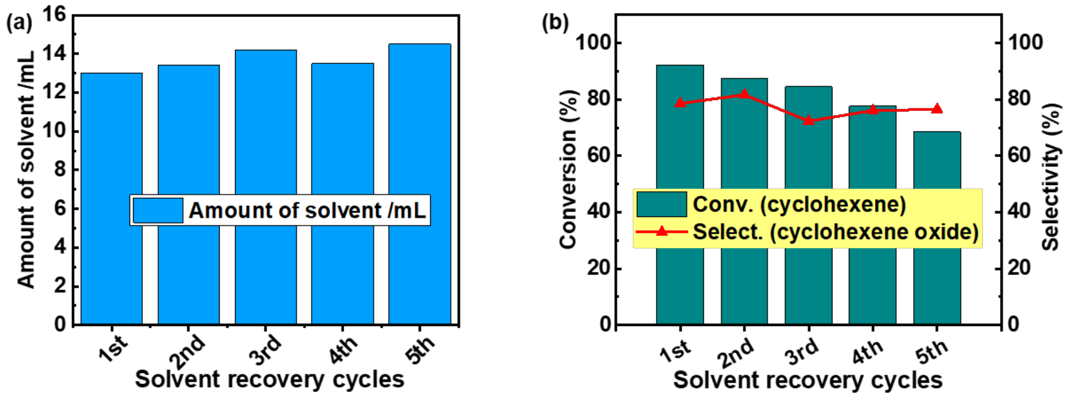

Figure S8. (a) Amount of solvent on the solvent recovery cycles;(b) The effect of solvent recovery cycles on the epoxidation of cyclohexene. Reaction conditions: cyclohexene (10 mmol), $MoSe_2$ (0.5 mmol), TBHP in decane(1 eq, 1.8 ml), HFIP (20 mL), 65 ℃，2 h. The conversions and selectivities were determined by GC with naphthalene as an internal standard. HFIP is recycled for 5 cycles.

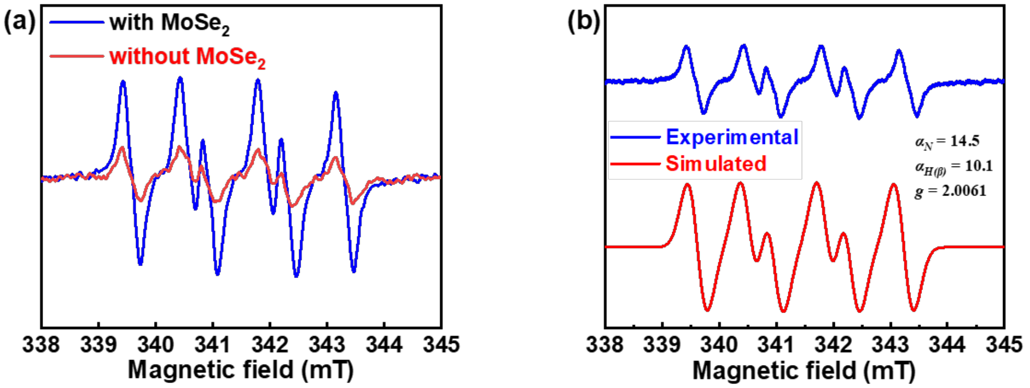

Figure S9. (a) ESR spectra of isobutene with TBHP and the spin trapping reagent (DMPO) in DCE at 75 ℃, blue: with $MoSe_2$, red: without $MoSe_2$. (b) EPR spectra and the corresponding simulation of isobutene with TBHP and the spin trapping reagent (DMPO) in DCE at 75 ℃.

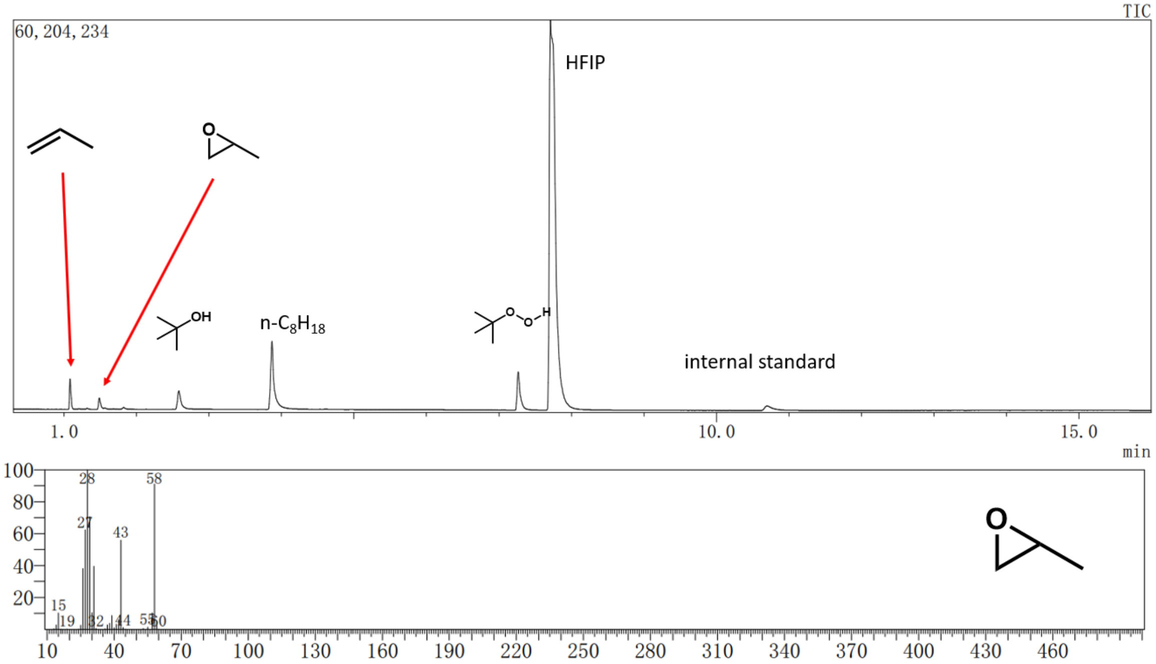

Figure S10. GC MS spectra of compound 2a

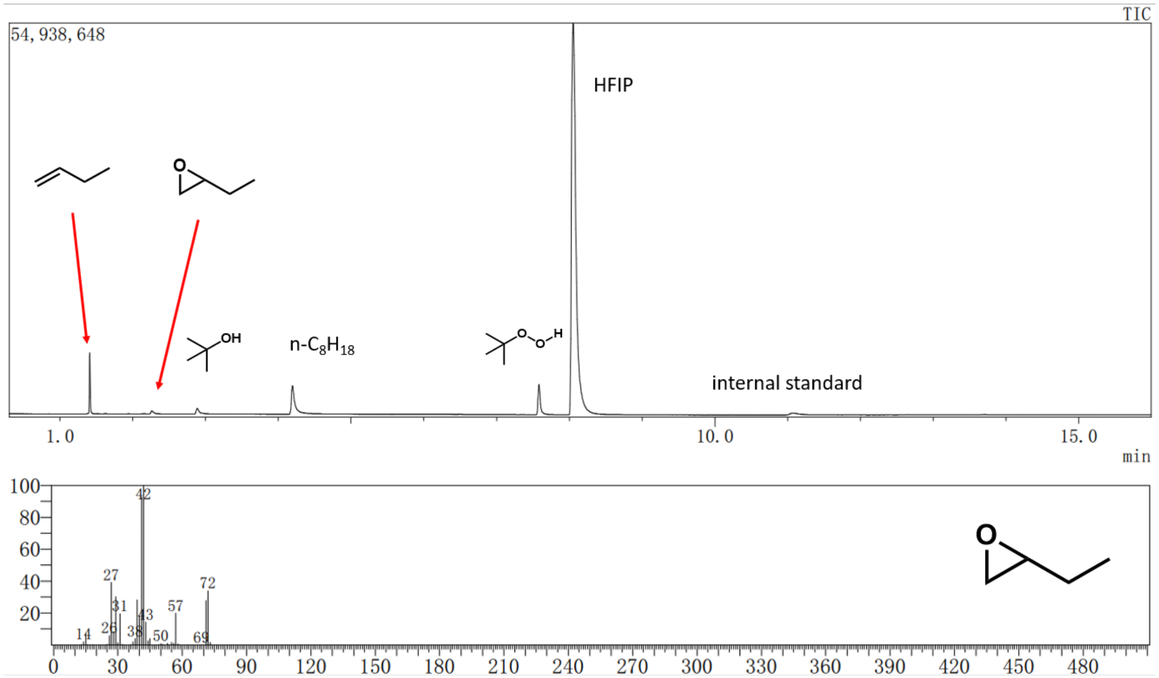

Figure S11. GC MS spectra of compound 2b

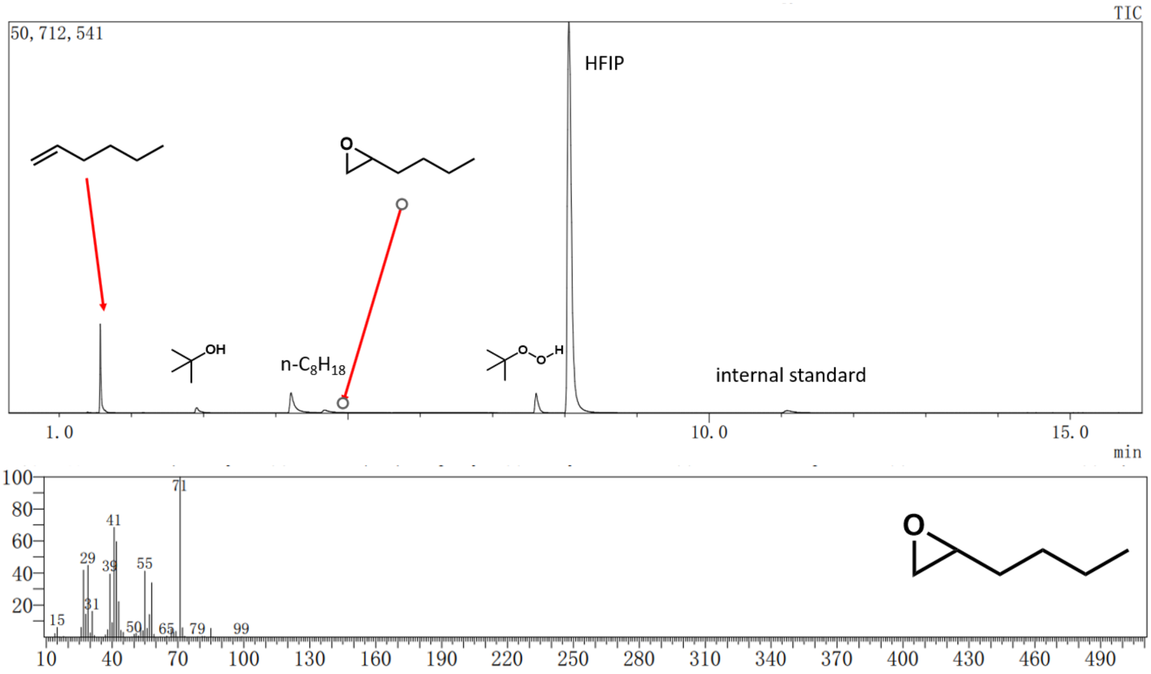

Figure S12. GC MS spectra of compound 2c

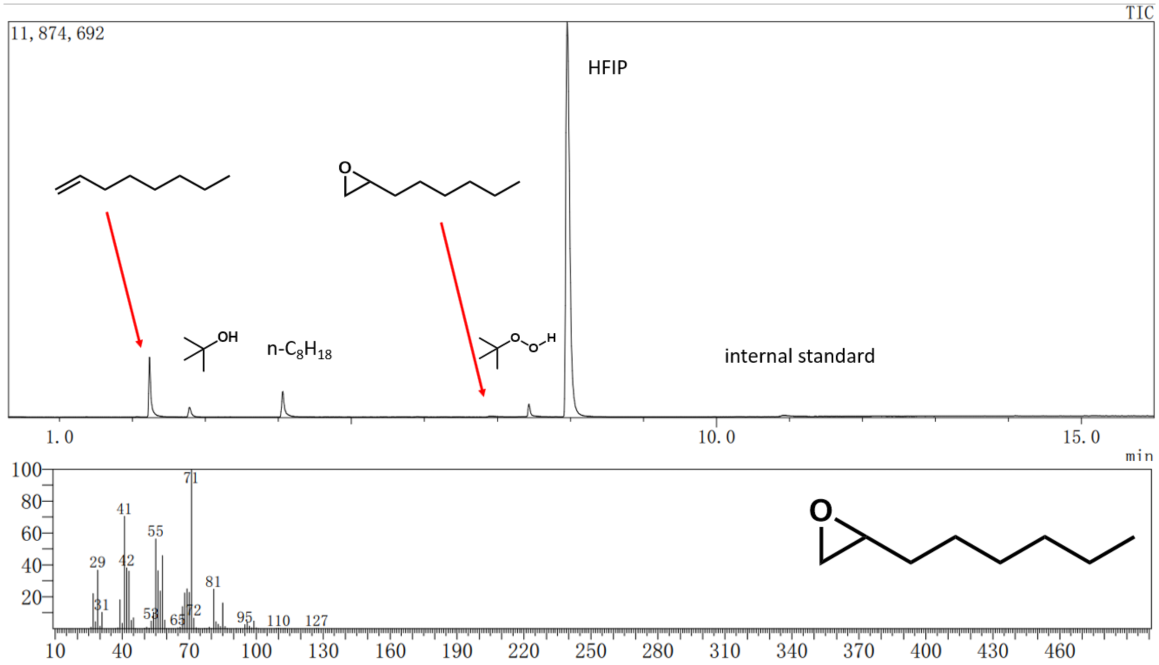

Figure S13. GC MS spectra of compound 2d

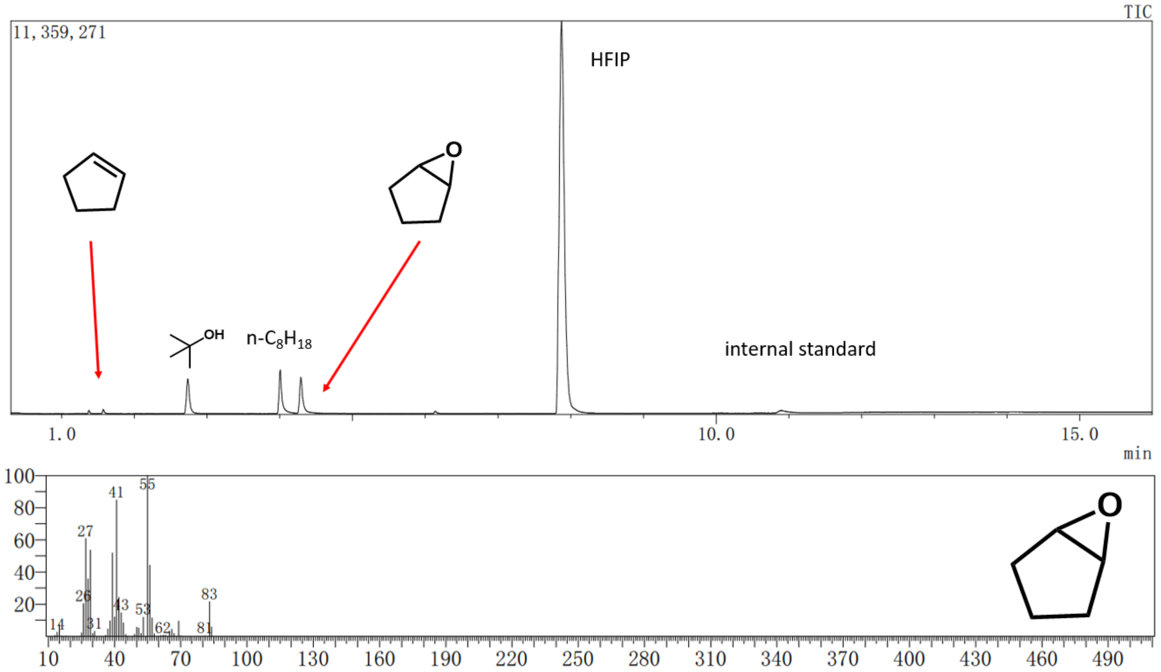

Figure S14. GC MS spectra of compound 2e

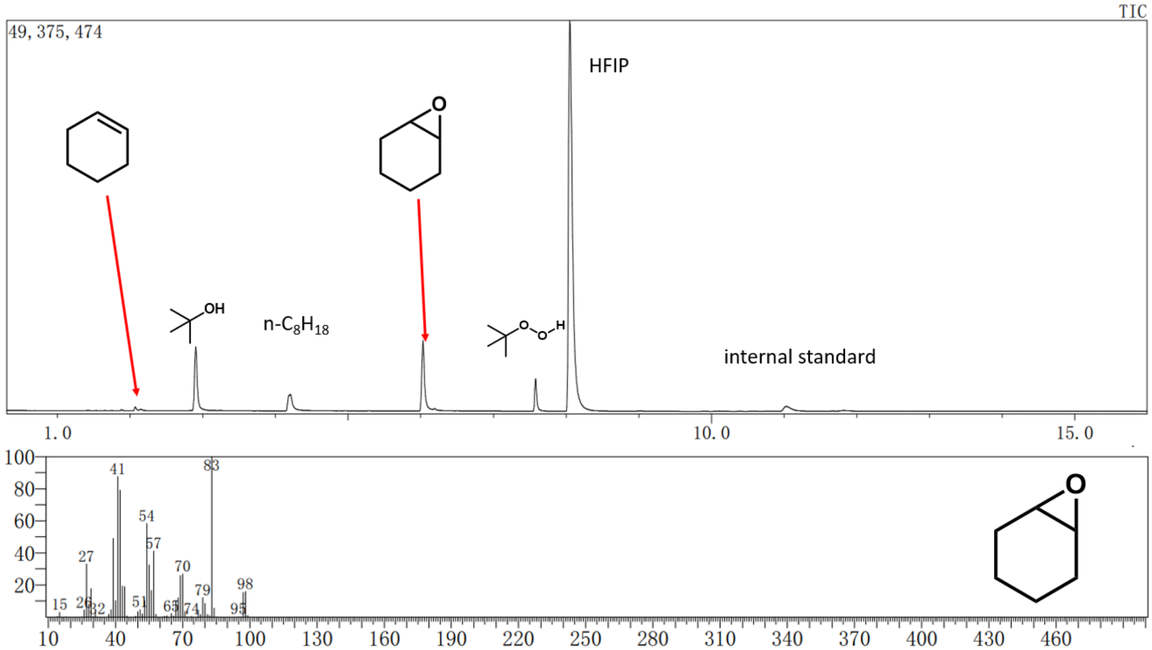

Figure S15. GC MS spectra of compound 2f

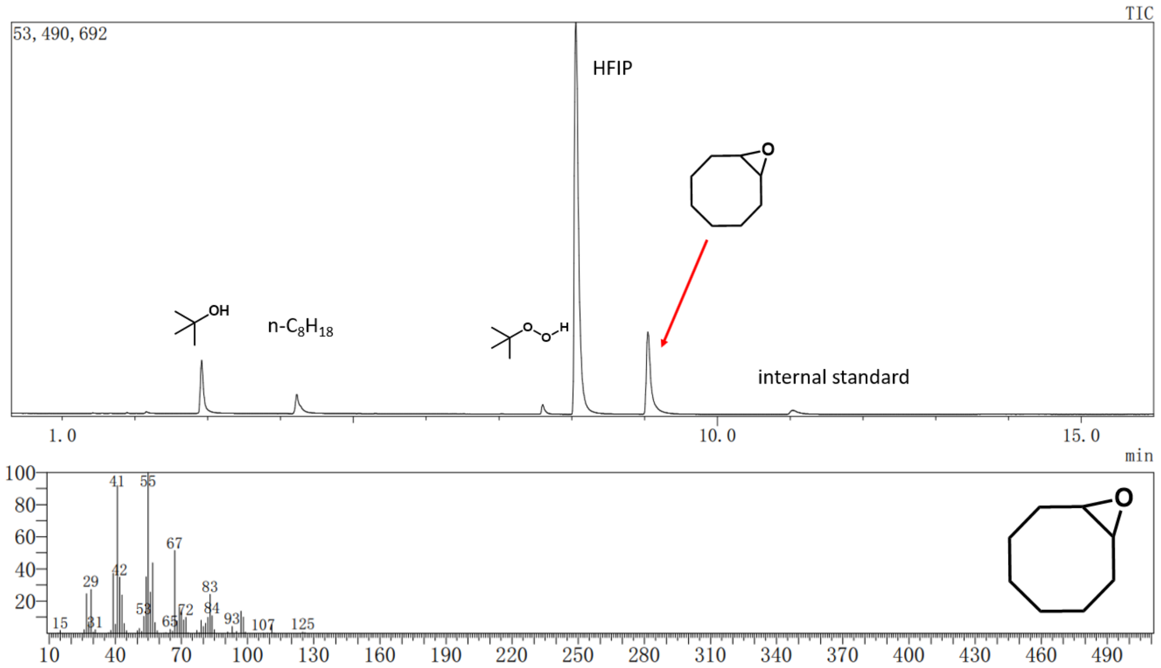

Figure S16. GC MS spectra of compound 2g

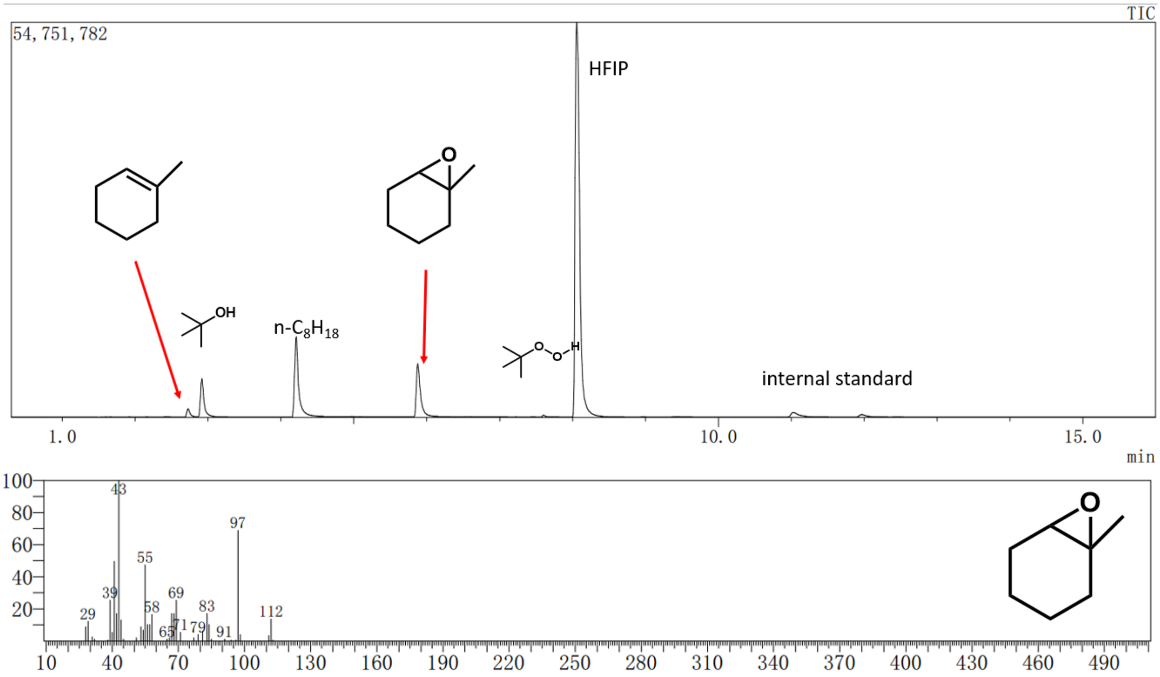

Figure S17. GC MS spectra of compound 2h

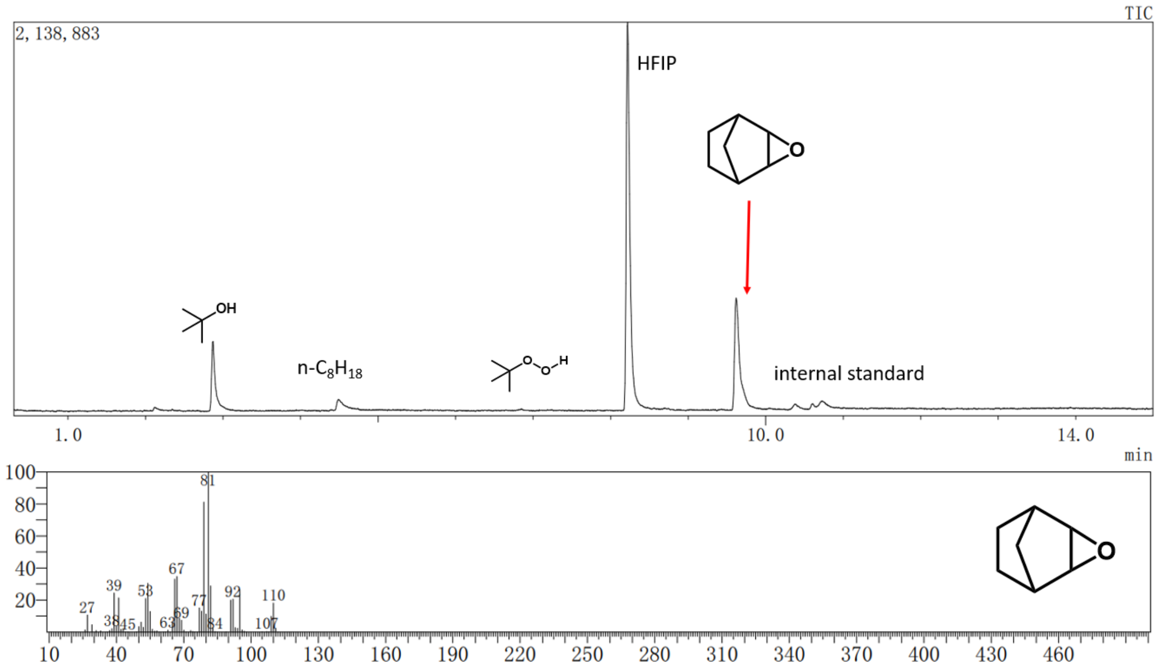

Figure S18. GC MS spectra of compound 2i

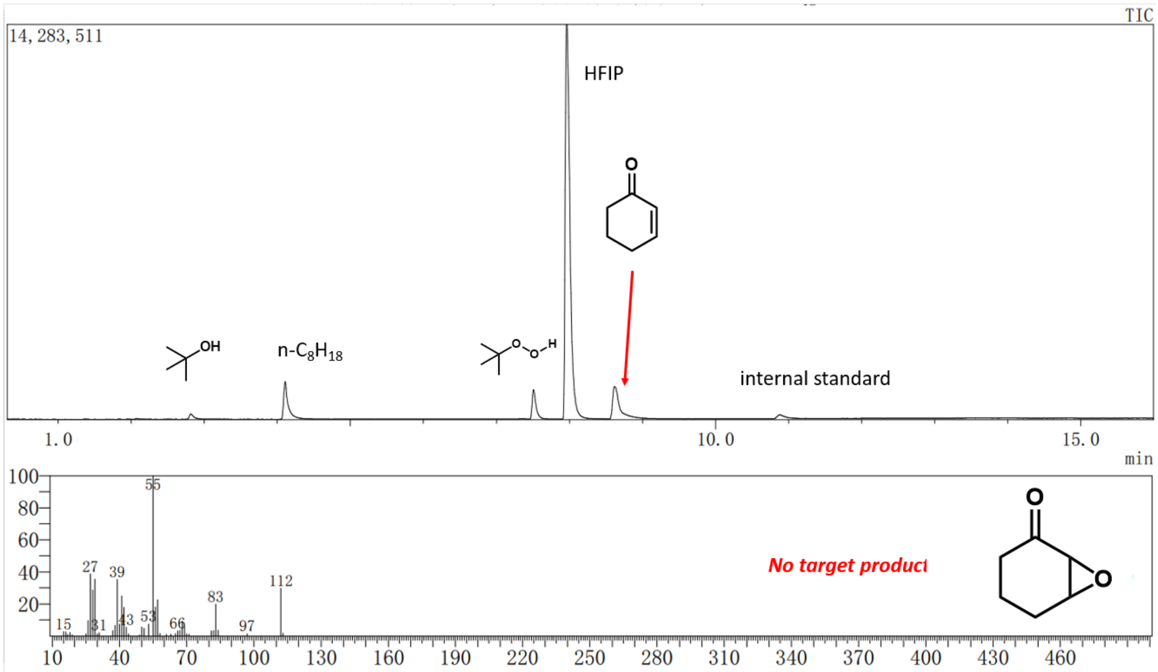

Figure S19. GC MS spectra of compound 2j

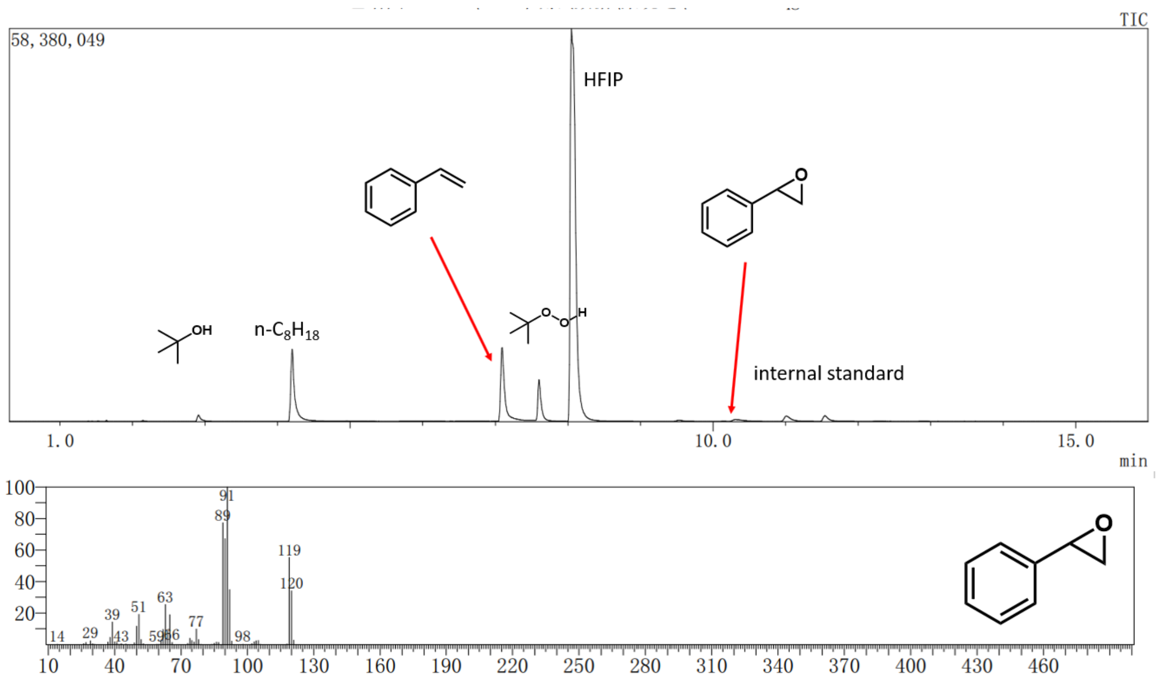

Figure S20. GC MS spectra of compound 2k

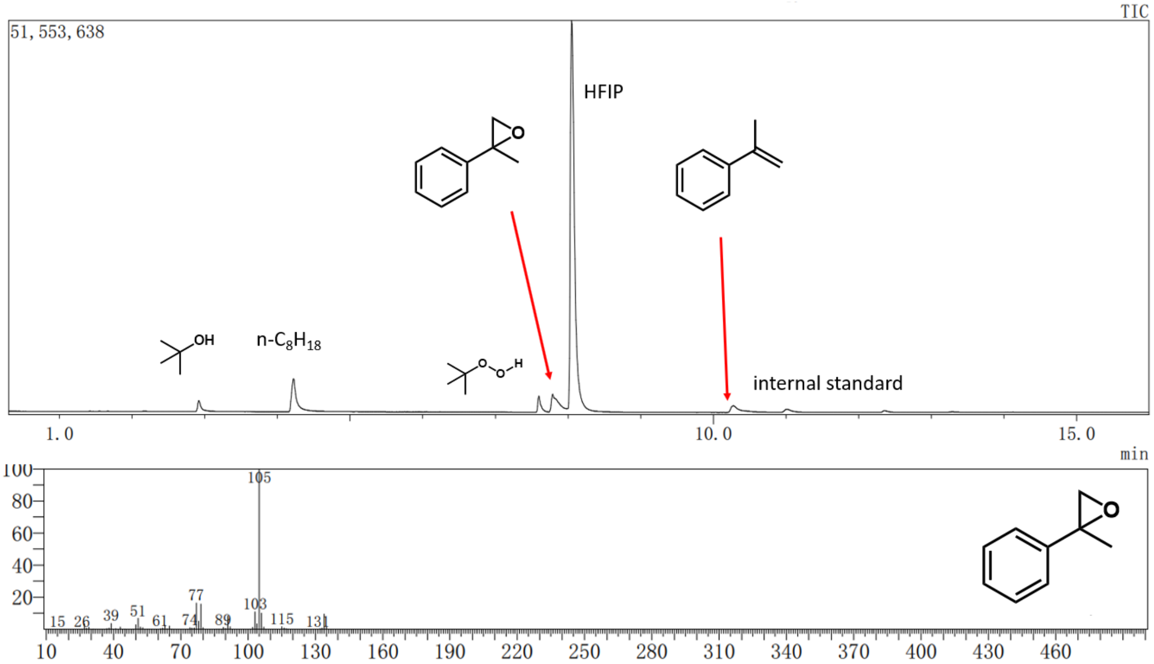

Figure S21. GC MS spectra of compound 2l
